# Development of a core outcome set for therapeutic clinical trials enrolling dogs with atopic dermatitis (COSCAD’18)

**DOI:** 10.1186/s12917-018-1569-y

**Published:** 2018-08-16

**Authors:** Thierry Olivry, Emmanuel Bensignor, Claude Favrot, Craig E. Griffin, Peter B. Hill, Ralf S. Mueller, Jon D. Plant, Hywel C. Williams, for the International Committee of Allergic Diseases of Animals (ICADA)

**Affiliations:** 1 Department of Clinical Sciences, College of Veterinary Medicine, North Carolina State University, 1060 William Moore Drive, Raleigh, NC 27606 USA; 2 Comparative Medicine Institute, North Carolina State University, Raleigh, North Carolina, USA; 3 Dermatology for Animals, Rennes, France; 4 Dermatology for Animals, Paris, France; 5 Dermatology for Animals, Nantes, France; 6 Vetsuisse Faculty University of Zurich, Clinic of Small Animal Internal Medicine, Zurich, Switzerland; 7 Animal Dermatology Clinic, San Diego, California, USA; 8 Companion Animal Health Centre, School of Animal and Veterinary Sciences, University of Adelaide, Roseworthy, South Australia Australia; 9 Medizinische Kleintierklinik, Centre for Clinical Veterinary Medicine, Ludwig-Maximilian University, Munich, Germany; 10 SkinVet Clinic, Lake Oswego, Oregon, USA; 11 Centre of Evidence-Based Dermatology, University of Nottingham Kings Meadow Campus, Lenton Lane, Nottingham, UK

**Keywords:** Allergy, Atopic dermatitis, Canine, Clinical trial, Core outcome set, Outcome measures

## Abstract

**Background:**

For decades, the efficacy of interventions in clinical trials enrolling dogs with atopic dermatitis (AD) relied on heterogeneous evaluations of skin lesions and pruritus using unvalidated tools. Although some instruments for clinical signs were validated later, there was little impact on standardizing outcome measures resulting in difficulties in comparing treatment efficacy between trials and impeding meta-analyses.

**Results:**

Participants in the Outcome Measures subcommittee of the International Committee of Allergic Diseases of Animals (ICADA) collaborated for two years to develop a core outcome set (COS) for canine AD, the COSCAD. This project involved several steps, constantly-re-assessed during online exchanges, to define the scope of this COS, to identify the relevant stakeholders, the domains to be evaluated, the instruments available for measuring agreed-upon domains and how to express outcome measures. This COSCAD’18 was designed principally for therapeutic—but not preventive or proactive—clinical trials enrolling dogs with chronic, nonseasonal (perennial), moderate-to-severe AD. Selected domains were skin lesions, pruritus manifestations and perception of treatment efficacy. Instruments to evaluate these domains were the CADESI4 or CADLI, the 10-point pruritus visual analog scale (PVAS10) and the Owner Global Assessment of Treatment Efficacy (OGATE), respectively. The COSCAD’18 has three outcome measures: the percentages of dogs with veterinarian-assessed skin lesions or owner-rated pruritus manifestation scores in the range of normal dogs or those with mild AD; the third is a good-to-excellent global assessment by the pet owners of their perception of treatment efficacy. Importantly, this COSCAD’18 is not meant to represent the sole—or primary—outcome measures evaluated in a trial; authors are always free to add any others, which they deem will best assess the efficacy of tested interventions. Benchmarks to define a threshold for treatment success were not set, as what constitutes a clinically-relevant therapeutic efficacy is expected to vary greatly depending interventions.

**Conclusions:**

This COSCAD’18 should help veterinarians and owners compare the benefits of treatments in future trials. This COS should also facilitate the combination of trial results in future systematic reviews, thereby producing more reliable summary estimates of treatment effects and enhancing evidence-based veterinary dermatology.

**Electronic supplementary material:**

The online version of this article (10.1186/s12917-018-1569-y) contains supplementary material, which is available to authorized users.

## Introduction

### Background

For the last three decades, clinicians have tested the efficacy of pharmacological and biological interventions to treat canine atopic dermatitis (AD). Three systematic reviews of clinical trial results—especially those performed in the last millennium—revealed that the therapeutic efficacy was assessed by outcome measures that varied greatly between investigating teams [1–3]. The domains evaluated in these trials were most often global assessments of the drug’s efficacy, or the assessment of one or more signs or symptoms.

At first, investigators used non-validated instruments (i.e., tools or scales) devised ad hoc or de novo to suit the various purposes. As a result, comparing the efficacy of the various therapeutic interventions was often unclear due to the profusion and variability of instruments and outcome measures used. Additionally, this inconsistency meant that one could not combine results in meaningful meta-analyses.

Since the beginning of this millennium, some instruments—or versions and variants thereof—were proposed in an attempt to standardize (or one could say “objectivize”) the evaluation of the cardinal signs of canine AD. The use of newly-validated instruments to measure the skin lesion and pruritus manifestation domains, for example the last two versions of the Canine Atopic Dermatitis Extent and Severity Index (CADESI3 or CADESI4) [4–6], the Canine Atopic Dermatitis Lesion Index (CADLI) [7] and Hill’s 10-point Pruritus Visual Analog Scale (PVAS10) [8, 9] was a notable improvement toward standardizing the evaluation of atopic dogs during trials. Unfortunately, there was still no universal consensus on which instruments to use, when and how to use them, and what would be considered an endpoint threshold for a clinically-relevant outcome or treatment “success”. Other authors took the liberty to transform these instruments by arbitrarily selecting and/or changing some of the evaluated lesions (i.e. the so-called “modified/mCADESIs”) [10, 11].

In recent trials used to support the approval of drugs for treatment of canine AD, clinicians most often used, as outcome measures, a 50% or greater reduction of baseline CADESI and PVAS values (i.e., the so-called CADESI-50, PVAS-50) [12–15] as a halving in the CADESI2 scores was shown to correlate highly with an overall assessment of treatment efficacy by both owners and clinicians [13]. Meanwhile, investigators also proposed a drug to be antipruritic if it led, *a minima*, to a 2-cm reduction out of a 10-cm PVAS [14]. Recently, in a large trial of a new therapeutic monoclonal antibody, the percentage reductions from baseline CADESI3 and PVAS values and the percentage of dogs whose endpoint CADESI3 and PVAS values were in the range of those of normal dogs were chosen [16]. As a result of such variability in selected outcome measures, the comparison of treatment effect between trials is still not always possible or even relevant.

In 2015, the International Committee of Allergic Diseases of Animals (ICADA) launched a subcommittee with the goal of proposing a “*core outcome set*” (COS) for clinical trials enrolling dogs with AD. Such COS would represent a small number of nonexclusive outcome measures, accepted by relevant stakeholders, to be assessed and reported in all clinical studies of similar design [17]. This COS would be a minimum set common to all trials. We wish to emphasize that a COS does not preclude investigators from measuring a host of other outcomes as needed for their study, nor does it mean that any of the COS measures need to be considered as a primary outcome measure.

In order to enhance our ability to compare and combine future therapeutic trials of canine AD, we sought to establish a COS for this disease. We report herein the development of the first COS in the field of veterinary dermatology: the 2018 Core Outcome Set for Canine Atopic Dermatitis (COSCAD’18) from the ICADA. Whenever applicable and relevant, this project was reported according to the recommendations of the Core Outcome Set-STAndards for Reporting (COS-STAR statement) [18].

## Methods

### Protocol/registry entry

The protocol was not registered beforehand due to the lack of relevant veterinary medicine registry for such purpose.

### Participants

We aimed to assemble a small group of ICADA members from either private specialty work or university practices, all with interest and experience in clinical trials and/or tool development for canine AD.

### Consensus process - design of the COSCAD’18

The design process of this COS evolved from addressing a series of questions in sequence; these were:

- Step 1: What is the scope of the COSCAD, i.e. for what type of patients and trials should this COS be designed?
- Step 2: What stakeholders would have to be consulted on the relevance of the chosen COS?
- Step 3: Which domains should be selected for evaluation in this COS?
- Step 4: Which instrument (s) should be used for each selected domain?
- Step 5: Which outcome measures using selected instruments should ultimately be included in this COS?
- Step 6: What would be desirable mode of reporting of the study COS?

Throughout the first half of 2016, subcommittee members worked on the successive steps of developments, via resources shared online, using a voting scheme that could be amended continuously while an issue was being reviewed.

During the de facto development phase of this COS (i.e. *steps 3 to 5*), all subcommittee members shared a file in which anybody could propose domains to be evaluated, the instruments to score these domains, and, ultimately, outcome measures using domain-instrument combinations. At the same time, each member had the constant opportunity to modify and vote on proposals made by others. Each individual step lasted until a consensus among at least 70% of members (i.e. five of seven) was reached on the specific points being discussed.

After the first draft of the COSCAD emerged, it was sent, in successive phases, to the stakeholders identified in the *Step 2* above using a combination of direct email exchanges and the completion of online forms. After each review phase, the proposed draft of the COSCAD was reassessed in the context of comments provided by the evaluators; this step provided an opportunity for an additional review and COSCAD modification before the proposal was sent out to the subsequent stakeholder group.

After this position paper was finally drafted, its content was agreed upon not only by all subcommittee member authors, but it was also approved unanimously by all ICADA members who reviewed it (18/18 members; 100%).

### Ethics and consent

We did not identify any relevant conflict of interest relevant to the development of this COS. While all subcommittee participants have received honoraria for consulting and/or lecturing or research support from commercial companies involved in animal health, this COS was not associated with any specific intervention or brand. Furthermore, all companies that had a drug approved specifically for treatment of canine AD were consulted in the third phase of stakeholder consultation. Three members of this subcommittee (PBH, TO, JP) had been involved in the development of the only validated instruments evaluating skin lesions [6, 7] and pruritus manifestations [9] in dogs with AD.

## Results

### Participants

Throughout the two years of development of this COSCAD’18, we had eight geographically-dispersed participants: Emmanuel Bensignor (specialty practice, multiple locations, France), Claude Favrot (University of Zurich, Switzerland), Craig Griffin (specialty practice, San Diego, California, USA), Peter Hill (University of Adelaide, Australia), Ralf Mueller (Ludwig-Maximilian University, Munich, Germany), Thierry Olivry (chair, NC State University, Raleigh, North Carolina, USA) and Jon Plant (specialty practice, Portland, Oregon).

### COSCAD development

#### Step 1: Scope

Participants reached a consensus that this COSCAD’18 should be proposed for all therapeutic—but not preventive, prophylactic or proactive—clinical trials enrolling dogs with chronic, nonseasonal (or perennial), moderate-to-severe AD.

A description of the methodology for diagnosing AD was beyond the purview of this subcommittee, but we expected that trialists enrolling subjects would diagnose AD mainly based on the classic clinical characteristics of this disease [19]. Furthermore, when developing this COS, we did not consider whether or not to evaluate dogs with environmental allergen-associated AD differently from those with food-induced AD or mixed IgE sensitization patterns [20], as these distinctions would have been made by investigators at the time of study design and atopic dog enrolment.

#### Step 2: Stakeholders

A consensus was reached to have evolving drafts of the COSCAD’18 reviewed, in successive phases, by all groups interested in the assessment of efficacy of interventions tested in clinical trials enrolling dogs with AD, as follows:

- *Phase I:* the entire membership of the ICADA.
- Phase II:
  - Board-certified veterinary dermatologists from existing specialty Colleges (American College of Veterinary Dermatology [ACVD], European College of Veterinary Dermatology [ECVD], Asian College of Veterinary Dermatology [AiCVD], and the Dermatology Chapter of the Australian and New Zealand College of Veterinary Scientists [ANZCVSc]).
  - atopic dog owners from various geographical areas.
- Phase III:
  - Representatives from the animal health companies that had at least one approved drug for treatment of canine AD.
  - Editors from the veterinary journals that had recently published articles reporting results of relevant clinical trials enrolling dogs with AD.
- Phase IV:
  - Representatives from the three largest administrative departments that have recently evaluated clinical trials for approval of drugs for treatment of canine AD in Europe and the USA (e.g. European Medication Agency (EMA), the Center for Veterinary Medicine of the US Food and Drug Administration [CVM-FDA] and the US Department of Agriculture [USDA]).

#### Step 3: Evaluated domains

The subcommittee members eventually reached a unanimous agreement to assess the following three domains for therapeutic clinical trials enrolling atopic dogs:

1. A veterinarian-reported assessment of skin lesions
2. An owner-reported assessment of pruritus manifestations (e.g. scratching, licking, rubbing, biting …)
3. An owner-reported global assessment of treatment efficacy

#### Step 4: Instruments for domain evaluation

The subcommittee members were unanimous in their selection of the following instruments to assess the three domains above:

1. *For the veterinarian-assessed skin lesions*: the 4th version of the Canine Atopic Dermatitis Extent and Severity Index (CADESI4) or the Canine Atopic Dermatitis Lesions Index (CADLI).
2. *For the owner-assessed pruritus manifestations*: the pruritus visual analog scale (PVAS) with category descriptors validated by Hill and Rybnicek with values translatable into a possible 10 points with a single decimal (PVAS10 from 0.0 to 10.0).
3. *For the owner-reported global assessment of treatment efficacy*: a subjective five-point Owner Global Assessment of Treatment Efficacy (OGATE) was proposed (Table 1).

**Table 1:** Owner Global Assessment of Treatment Efficacy (OGATE)

Question: How would you rate the overall response to treatment?	
Answer: select only one of the following five answers:	
0 - No response	
1 – a poor response	
2 – a fair response	
3 – a good response	
4 – an excellent response	

The first three instruments (CADESI4/CADLI/PVAS) were identified easily, as these are the only ones with published reports of a combination of either validity, reliability, sensitivity to change or thresholds for severity levels [6–9]; these tools were logical choices due to their popularity and use in clinical trials for many years. While we considered the time to administer the skin lesion-grading instrument in our evaluation processs, the limited time needed to grade skin lesions with either CADESI4 and CADLI (both with median grading times of less than 5 min) makes them easy to use when seeing patients, and they were considered equivalent in this regards [6].

The subcommittee members do recognize, however, that the OGATE is not a validated instrument, but at the time of development of this COSCAD, a validated global “*patient-oriented outcome measure*” could not be identified in any previously-published clinical trials identified in the three available systematic reviews [1–3]. Notwithstanding this caveat, the subcommittee members unanimously agreed that such OGATE would capture the owner’s perception of the benefit of the proposed treatment, a parameter of highest importance as it would have some implications on the adherence to the prescribed regimen and the quality of life of the patient and its owner (s).

#### Step 5: Use of selected instruments as outcome measures in the COSCAD’18

While authors of articles reporting results of clinical trials enrolling dogs with AD will naturally be free to use any one or combination of outcome measures of their choice, the members of this subcommittee recommend that reported efficacy measures for relevant trials include **at least** the COS below.

This COSCAD’18 is composed of the following three outcome measures, which should be reported for each tested intervention, be it an active one, a placebo, a single drug or a combination regimen:

1. CADESI4/CADLI (normal-to-mild)
  This outcome measure, to be abbreviated CADESI4-N2M or CADLI-N2M, corresponds to the percentage of dogs with veterinarian-assessed skin lesion scores in the range of normal dogs or those with mild AD at study end (i.e. dogs with CADESI4 < 35 or CADLI < 8).
  (Example of future reporting: X% of the dogs had skin lesion scores of normal dogs or those with mild AD after being treated with drug Y for Z weeks)
2. PVAS10 (normal-to-mild)
  This outcome measure, to be abbreviated PVAS10-N2M, tallies the percentage of dogs with owner-assessed pruritus manifestation scores in the range of normal dogs or those with mild AD at study end (i.e. dogs with PVAS10 < 3.6).
  Example of future reporting: X% of the dogs had pruritus manifestation levels of normal dogs or those with mild AD after being treated with drug Y for Z weeks.
3. OGATE (good-to-excellent)
  This outcome measure, to be abbreviated OGATE-G2E, represents the percentage of dogs whose owner rated the overall response to treatment as “*good*” or “*excellent*” (i.e. OGATE > 2).
  Example of future reporting: X% of owners evaluated that treatment with the drug Y for Z weeks had a good-to-excellent response.

Readers should be mindful that this COSCAD’18 was designed for dogs with **moderate-to-severe AD** at the time of enrolment in a clinical trial. In case the investigators were to wish to enroll dogs with skin lesions and/or pruritus manifestations representing **mild** AD, then the two primary outcome measures would have to be changed to *“… scores in the range of those of normal dogs*”. The CADESI4-N, CADLI-N and PVAS10-N threshold values would then change to less than 10, 6 and 2.0, respectively.

Furthermore, researchers should be conscious that the CADESI4 and CADLI evaluate, among others, some skin lesions (e.g. lichenification and self-induced alopecia) that will not be sensitive to change in short-term trials lasting less than 6 weeks. As a result, this COSCAD’18 will be most relevant and representative of the true intervention’s efficacy in **trials lasting 6 weeks or longer**.

Finally, and most importantly, while this COSCAD’18 is designed to include three separate outcome measures to be used in clinical trials, it does not define benchmarks that would constitute a clinically-relevant treatment success by themselves. Indeed, such assessment is likely to vary depending upon the potency and characteristics of the intervention tested. As a result, this subcommittee leaves the approving authorities in the various countries to define what percentage of atopic dogs enrolled in the various trials coming under their review should meet which benchmark as a primary efficacy measure; the selected outcome measures could be among those included in the COSCAD’18, or they could be any other.

Further details on the four phases of evaluation of the COSCAD’18 by the different stakeholders is available online as Additional file 1.

#### Step 6: Recommendations for data reporting

There was consensus among subcommittee members that, to have a better depiction of the treatment effect, the reporting of study results in journal articles should have, in addition to the COSCAD’18, a minimum degree of standardization.

We recommend the reporting to include, ideally, all of the following:

1. a comprehensive online supplementary table with all individual subjects pre- and post-treatment CADESI4/CADLI and PVAS10 values and post-treatment OGATE (Additional file 2).
2. an online supplementary or in-article published scatter plot figures with all pre- and post-treatment CADESI4/CADLI and PVAS10 (Additional file 3), and
3. a published table reporting the number and percentage of dogs with CADESI4/CADLI and PVAS10 values in the different severity categories at the various evaluation time points (*a minima* those before and after treatment [Additional file 4]).

## Discussion

### Main findings

In this paper, the Outcome Measures Subcommittee of the ICADA reports the design of the COSCAD’18, the first COS for therapeutic trials enrolling dogs with nonseasonal moderate-to-severe AD. This COSCAD’18 contains three outcome measures.

The first two evaluate domains (skin lesions and pruritus manifestations) and use validated instruments that are already widely-known to the veterinary dermatology community. At this time, veterinarians are familiar with the reporting of percentage changes from baseline CADESI/CADLI/PVAS10 values during trials. While a 50% reduction in values (e.g. a CADESI-50) has been used for over 15 years [12, 13], members of this subcommittee were not supportive of using such outcome measure because of its dependency upon baseline values resulting in heterogeneous reductions in the score themselves. For example, a treatment-induced 50% reduction in a severely itchy dog with a baseline PVAS10 of 10.0 corresponds to a 5-point grade reduction and a residual score corresponding to a moderate pruritus level likely to be found unacceptable by most owners. In contrast, the same 50% reduction in a dog with a moderate pruritus level and a baseline PVAS10 of 3.8 will lead to a 1.9 grade change and an ending level of pruritus corresponding to that of a normal dog.

As a result, instead of percentage changes from baseline scores (the so-called “*deltas*”), we preferred to have the COSCAD’18 include the percentage of dogs having skin lesion and pruritus manifestation scores in the range of those of normal dogs or those with mild AD at trial’s end. It was felt that, ultimately, the veterinarians and dog owners would prefer to know how likely the treatment would make their patient/pet looking normal or having only residual mild signs and symptoms of AD.

As a third outcome measure, we proposed to use the OGATE, an owner-assessed global evaluation of their perception of the efficacy of the treatment used in their pet. While this instrument has not been formally validated per se, the subcommittee participants felt that such an evaluation was one that would matter most to pet owners, as it would influence treatment adherence and the quality of life of their pet. We wish to point out, however, that a close ancestor of this OGATE—then named the OGA-E, “E” for “Efficacy”—had been first proposed as part of the evaluation of the sensitivity to change of the CADESI4 [6]; the OGA-E was significantly and highly correlated with the percentage reduction from baseline of the CADESI4 after a therapeutic intervention [6]. This observation suggests that the higher the post-treatment reduction in skin lesion scores, the higher the owner’s assessment of efficacy would be of such treatment, a logical and clinically-relevant expectation.

### Limitations

The main limitation in the design of this COSCAD’18 is the relatively small size of the communitiy involved in its development and stakeholder validation, which could be perceived as a source of bias. While it would have been ideal to involve all identified stakeholders in the early selection of the domains, tools and outcome measures, the global audience targeted and the lack of independent financial resources to assemble large audiences were deterrents to such achievement. Nevertheless, each successive draft of the COSCAD’18 was influenced by the feedback of the preceding stakeholder groups. Indeed, each online poll had a comment box for surveyed individuals to leave a feedback including additional outcome measures. Additionally, during the later phases of the stakeholder consultation (*Phases III and IV*), consultees were allowed more time and they did not have to use a predefined format; most answered in a lengthier fashion documenting the rationale of their evaluation.

A second limitation is our proposed use of the formally-unvalidated OGATE and its perception of subjectivity that contrasts with that of a more “*objective*” assessment of lesion and pruritus manifestation scores with the CADESI4/CADLI and PVAS10, respectively. While this subcommittee’s participants agreed that the OGATE is in need of a proper validation, readers should keep in mind that the CADESI4 and CADLI merely represent aggregated subjective scores of individual signs or, for the PVAS10, symptoms; the OGATE should then only be seen as a similar subjective evaluation of a perception of treatment efficacy.

### Implications for research

There are several research needs that derive from the development of this COSCAD’18.

Firstly, the subcommittee participants could not decide which one of the CADESI4 or CADLI should be preferred, as both were found to evaluate the same domains, lesions, and their scores have been shown to correlate highly; a significant 86% correlation was found between CADESI4 and CADLI values in [6]. In spite of these two scoring systems being deemed equivalent, further studies should determine which one might perform best in atopic dogs of varying severities, so that future COSCAD updates only propose one of these instruments for simplicity purposes.

Secondly, the OGATE itself should be evaluated, *a minima*, for its validity and reliabilty, as described previously [21]. Its sensitivity to change between weak and potent interventions, and between short and longer courses thereof, must also be quantified.

During the process of domain selection, the participants in this subcommittee considered including “quality of life (QoL)” as a parameter. While there are two instruments proposed for the evaluation of the QoL in dogs with skin diseases [22–25], the subcommittee members considered these instruments overtly complicated and in need of further simplification and ensuing validation.

Finally, in most recent trials testing interventions in humans with AD, outcome measures have often included an “Investigator Global Assessment (IGA)” of “clear-or-almost clear”. While there is no universal agreement on a standard and validated instrument to assess this IGA [26], such an outcome measure would likely be highly relevant in trials enrolling dogs with AD. Indeed, an IGA could serve to increase the validity of the assessments of two of the COSCAD’18 outcome measures with which the IGA would be expected to correlate: the CADESI4/CADLI-N2M and the PVAS10-N2M. As there is currently no IGA available for use in dogs with AD, the design and validation of such an instrument could be a valuable tool to be used in future trials.

### Implications for practice

While the various instruments discussed above were designed for their use in clinical trials enrolling dogs with AD, it is possible that one or more—especially the simple OGATE—could be useful to veterinarians in the follow-up of treatment of their patient dogs with AD. Such usefulness needs to be tested appropriately.

## Conclusion

With this COSCAD’18, the Outcome Measure Subcommittee of the ICADA hopes to provide a new standard for the standardization of the reporting of clinical trials enrolling dogs with AD. Our aim is not to stifle investigator independence, but to harmonise study outcomes in order to make a better sense of future studies. Study authors, whether animal health company employees or independent investigators, are encouraged to include this COSCAD’18, either as a stand-alone outcome set or as part of their trials’ selected outcome measures. It is also hoped that other relevant stakeholders (drug approval authorities, journal editors and article reviewers) will work together to generalize the use of this COSCAD’18. Hopefully, this will allow the entire veterinary community, and most importantly the pet owners, to be better prepared in their shared decision making when comparing the various interventions available to treat dogs with AD.

## Additional files

Additional file 1: This file contains the specific data corresponding to the evaluation of the proposed COSCAD’18 by the various stakeholders. (DOCX 137 kb)

Additional file 2: a. Example of desirable comprehensive online supplementary table with all individual subjects pre- and post-treatment COSCAD’18 values. b. Example of desirable tables for reporting the COSCAD’18 with possible relevant statistical analyses. (XLSX 51 kb)

Additional file 3: a. Example of desirable scatter plot figure. b. Example of desirable after-vs-before figure. (TIF 1258 kb)

Additional file 4: Example of in-article table with data categorization and possible relevant statistical analyses. (XLSX 32 kb)

## Abbreviations

AD: Atopic dermatitis
COS: Core outcome set
COSCAD: Core outcome set for canine atopic dermatitis
ICADA: International Committee for Allergic Diseases of Animals
RCT: Randomized controlled trials
