# Supplementary material for: Development of a core outcome set for therapeutic clinical trials enrolling dogs with atopic dermatitis (COSCAD’18)
Source: BMC Vet Res. 2018 Aug 16;14:238. doi: 10.1186/s12917-018-1569-y (PMC6097451; doi:10.1186/s12917-018-1569-y)
Supplement: Supplementary file 1 — This file contains the specific data corresponding to the evaluation of the proposed COSCAD’18 by the various stakeholders. (DOCX 137 kb) [file 12917_2018_1569_MOESM1_ESM.docx]

***Stakeholder Evaluation of the COSCAD’18***

*Phase I:*

- The entire membership of the ICADA: By January 6, 2017, 100% of the 20 ICADA members that expressed their opinion had rated each of the three COSCAD’18 outcome measures to be relevant.

*Phase II:*

- Veterinary dermatologists: By February 15, 2017, between 86 and 96% of 130 surveyed European and American board-certified veterinary dermatologists found the three COSCAD’18 outcome measures to be relevant.
- Atopic dog owners: Between April and June 2017, 110 owners from dogs with AD were surveyed; they came from 19 countries and five continents. Owners were nearly unanimous (100 and 99%) in their assessment that the proposed outcome measures would help them understand whether the intervention tested was genuinely effective.

*Phase III:*

- Animal health companies*:* representatives of the three companies that have at least one approved drug for treatment of canine AD were sent the proposed COS for their review in the summer of 2017. These companies provided detailed comments, which were further reviewed by this committee in the Fall of 2017. Modifications in the scope of this COS and some limitations of the proposed instruments were later included in the final version of the COSCAD’18.
- Journal editors*:* the editors of three journals having recently published clinical trials enrolling dogs with AD were asked to comment on the proposed COS. Only one response was received, which provided no further comments.

*Phase IV:*

- Drug approval administrations: Feedback was received from veterinarians employed by the three administrations approving drugs for treatment of canine AD in Europe and the USA. One administrator explained their department’s philosophy not to be involved in such a process. Another confirmed the relevance of the scales used, but a preference for single lesion ratings (e.g. erythema) over composite scales (e.g. CADESI4). Finally, the third representative found the COSCAD’18 to be relevant with a preference for the outcome measures that use validated instruments (CADESI4, CADLI and PVAS10) over the unvalidated OGATE, the latter likely being preferred as a secondary rather than a primary outcome measure. Our committee examined these comments and decided to keep recommending the composite instruments CADLI and CADESI, as only the aggregated scales, but not the separate lesion scores, have been validated.
