# Supplementary figures and images for: Development of a core outcome set for therapeutic clinical trials enrolling dogs with atopic dermatitis (COSCAD’18)
Source: BMC Vet Res. 2018 Aug 16;14:238. doi: 10.1186/s12917-018-1569-y (PMC6097451; doi:10.1186/s12917-018-1569-y)

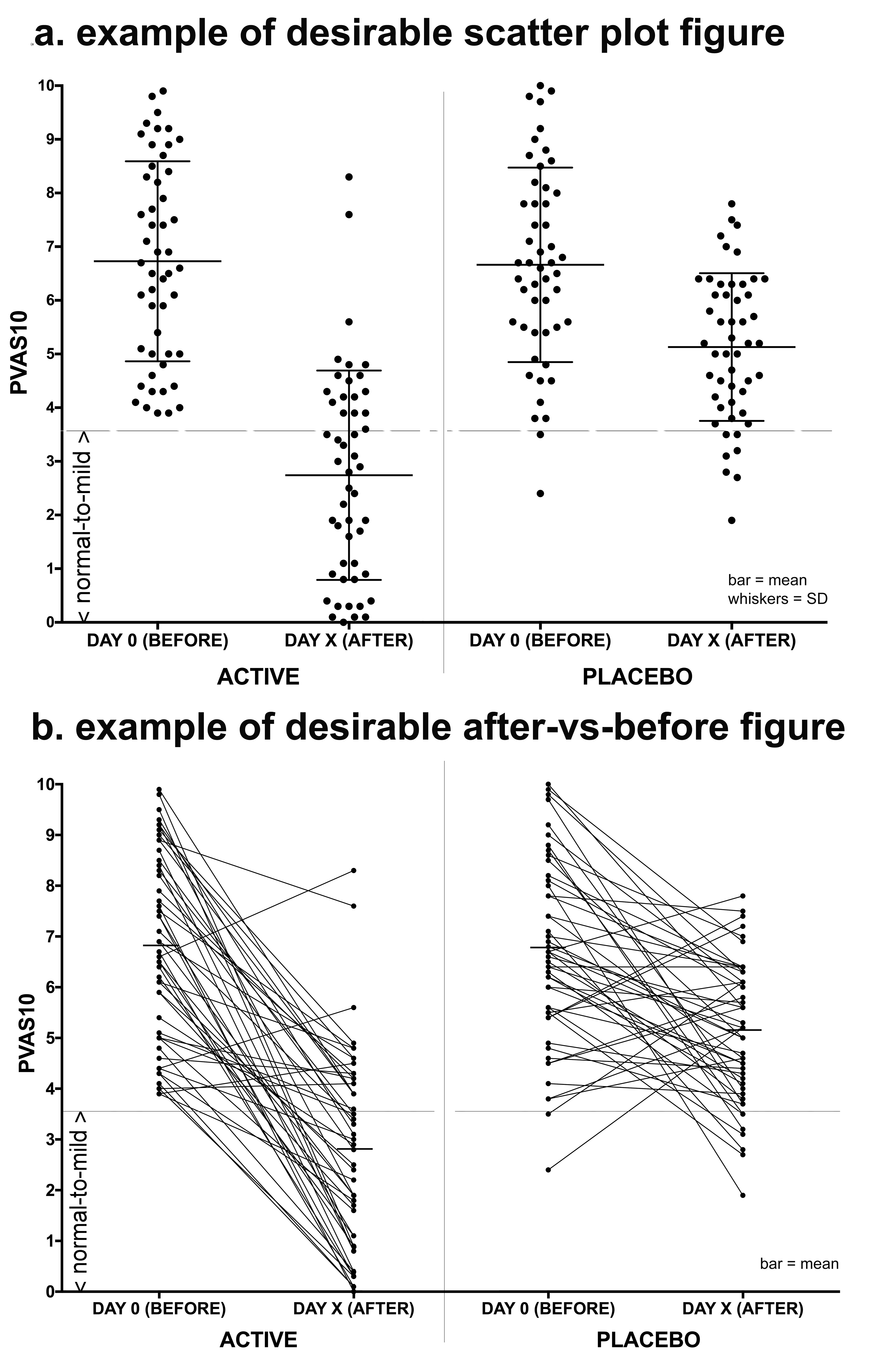

Supplement: Supplementary file 3 — a. Example of desirable scatter plot figure. b. Example of desirable after-vs-before figure. (TIF 1258 kb) [file 12917_2018_1569_MOESM3_ESM.tif]
